# Editorial: Targeting the Immune System to Treat Hepatitis B Virus Infection

**DOI:** 10.3389/fimmu.2022.868616

**Published:** 2022-03-15

**Authors:** Zhongji Meng, Jia Liu, Anna D. Kosinska, Mengji Lu

**Affiliations:** ^1^ Institute of Biomedical Research, Hubei Clinical Research Center for Precise Diagnosis and Therapy of Liver Cancer, Taihe Hospital, Hubei University of Medicine, Shiyan, China; ^2^ Department of Infectious Diseases, Union Hospital, Tongji Medical College, Huazhong University of Science and Technology, Wuhan, China; ^3^ Institute of Virology, Technische Universität München/Helmholtz Zentrum München, Munich, Germany; ^4^ Institute of Virology, University Hospital of Essen, University of Duisburg-Essen, Essen, Germany; ^5^ German Center for Infection Research (DZIF), Munich Partner Site, Munich, Germany

**Keywords:** hepatitis B Virus, Immune response, immune tolerance, immunotherapy, T cell therapy, innate immunity, therapeutic vaccine

The dysfunctional immune responses play an essential role in persistent hepatitis B virus (HBV) infection as well as in liver inflammation. Modulation of the host immunity to strengthen specific cellular immune responses might help control HBV infection. Various strategies to restore and enhance innate and HBV-specific adaptive immune responses have been tested in preclinical studies and clinical trials and some with significant antiviral effects. However, the molecular mechanisms accounting for HBV persistence are still not fully elucidated despite of great progress in the research field. The current Research Topic issue covers a wide range of subjects in the immunopathogenesis and immune regulation in chronic HBV infection.

Interferons (IFNs) play diverse crucial roles in both innate and adaptive immune responses due to its ability to exert direct antiviral and immune regulatory functions. IFN-α or pegylated IFN-α has been widely used in the treatment of chronic hepatitis B (CHB) with the potential of functional cure, especially in patients with low level of HBsAg and viral loads. Ye and Chen summarize the status and unique advantages of IFN therapy against CHB, including the mechanisms of IFN-α action and factors affecting IFN response, and the options for improvement of IFN-based therapy and the rationale of combinations with other antiviral agents to achieve an HBV cure. Sajid et al. showed in their study that the overexpression of an IFN-α inducible gene IFI6 could inhibit HBV replication and gene expression in hepatoma cells and in the hydrodynamic injection (HDI) mouse model. The study added a new piece to the molecular mechanism of IFN-mediated antiviral functions.

Pathogen recognition receptors (PRRs) are key components in the host immunity through recognizing various conserved molecular patterns of pathogens and activating innate and adaptive immune responses. Immune modulation targeting innate immunity for the treatment of CHB has attract attentions in recent years. Suresh et al. investigated the expression kinetics of receptors from various PRR families (RLRs, NLRs, TLRs, CDSs, and inflammasomes) during acute, self-limited woodchuck hepatitis virus (WHV) infection. The study demonstrated differential intrahepatic expression of PRRs and immune cell markers during acute self-limiting infection and the progression to persistent WHV infection. Their results suggested that a weak innate immune response, most likely by type-I IFN production via activated viral DNA and selected RNA sensing receptor pathways, occurred in woodchucks with the progression to chronic infection, while the adaptive immune response was largely absent. In another paper, Suresh et al. demonstrated that, liver-targeted delivery of poly(dA:dT) induced the intrahepatic expression of ZBP1/DAI and AIM2 receptors and their effector cytokines, IFN-β and interleukins 1β and 18. The antiviral effect on WHV replication and production following *in vitro* activation of IFI16, ZBP1/DAI, and AIM2 receptor pathways was improved by targeting more than one cytosolic DNA receptor. The liver-targeted delivery of PRR agonists may improve the therapeutic efficacy against HBV. Ayithan et al. reported the effect of TLR8 agonism on supporting cytokines and follicular helper T (T_FH_) and B cells in their recent study. Their results demonstrated that triggering TLR8 could induce IL-12 production in monocytes, which in turn leads to differentiation of CD4+ T cells into IL-21 producing T_FH_ in peripheral blood samples from CHB patients. Accordingly, co-culture of these differentiated T_FH_ with autologous B cells resulted in B cell differentiation into plasma cells and promoted IgG production. Finally, improved HBsAg-specific T_FH_ and B cell responses were observed in a fraction of CHB patients treated with a selective TLR8 agonist. The study suggested that TLR8 signaling has potential to repair a critical defect in T-B interaction that is necessary for robust B cell response.

Chronic HBV infection is characterized by quantitatively and qualitatively weak HBV-specific CD8+ T cell responses. To define the mechanisms of HBV-Specific CD8+ T cell dysfunction, Baudi et al. summarized data about the HBV-specific CD8+ T-cell tolerance in the liver, including immunoregulatory mediators in liver tolerance, negative signaling mechanisms, metabolic dysregulation in T cells, and intrahepatic antigen recognition. The elucidation of the key pathways and processes underline HBV-specific CD8+ T cell dysfunction will facilitate the development of effective HBV immunotherapies. Wang et al. reported in their recent work that, CXCR3+CXCR6+ γδT cells produced high levels of IFN-γ during acute HBV infection. An adoptive transfer of CXCR3+CXCR6+ γδT cells into acute HBV infected TCRδ-/- mice led to reduced HBsAg and HBeAg expression. Their work suggested that liver resident CXCR3+CXCR6+ γδT cells play a protective role during acute HBV infection. The role of CXCR3+CXCR6+ γδT cells in immunotherapy for chronic HBV infection needs further study.

Identification of immunogenic targets against HBV-encoded proteins will provide crucial advances in developing potential antibody therapies. Gu et al. performed a screening on B-cell linear epitopes with sera from patients in different phases of the natural history of chronic HBV infection. Their study found that dominant linear B-cell epitopes are expanded in CHB. The proportion of dysfunctional atypical memory B cells was decreased in patients who achieved HBsAg loss and associated with successful treatment withdrawal. In this study, 7 dominant epitopes were recognized by antibodies from patients with HBsAg loss. The baseline of antibodies to a specific HBsAg epitope was found to be associated with a favorable treatment response to telbivudine therapy. Future studies are needed to confirm the diagnostic values of antibodies to linear epitopes of HBV proteins.

A functional cure for chronic HBV infection could be achieved by boosting HBV-specific immunity. HBV-specific chimeric antigen receptor T (CAR-T)/T-cell receptor T (TCR-T) cells are promising therapeutic approaches for treatment of CHB, but with potential risks of inducing cytokine release syndrome or hepatotoxicity. Klopp et al. generated an inducible caspase 9 (iC9) as a safety switch to control HBV-specific S-CART or TCR-T. *In vivo*, induction of iC9 in S-CAR T cells resulted in a strong and fast depletion of transferred T cells, leading to the loss of antiviral efficacy and preventing liver toxicity and cytokine release at the same time. Ferrando-Martinez et al. found that PD-L1 blockade could enhance the HBV-specific CD8 T cell response only in patients with lower frequencies of functionally exhausted HBV-specific CD8 T cells as indicated by a phenotype of LAG3+TIM3+PD-1+. Higher levels of functionally exhausted HBV-specific CD8 T cells in CHB patients are associated with a lack of response to the anti-PD-L1 antibodies. Thus, blocking the PD-1:PD-L1 axis as a monotherapy may only have limited clinical effectiveness. Combination strategies with antibodies to other anti-inhibitory receptors like LAG-3, will likely be required to elicit a functional cure for patients with high levels of functionally exhausted HBV-specific CD8 T cells.

The NF-κB signaling pathway plays a key role in the development and function of host immune system and in inflammation and viral infection. Lu et al. summarized the knowledge about the interplay between non-canonical NF-κB signaling and HBV infection. They came to a conclusion that the non-canonical NF-κB signaling pathway plays also an important role in triggering inflammation in HBV-related diseases, while the mechanism of the interplay between HBV and non-canonical NF-κB signaling is largely unknown and needs further in-depth investigation.

The cellular mechanisms underlying the non-responsiveness of HBV vaccination are still poorly understood. Körber et al. reported significantly higher frequencies of CD24^high^CD38^high^ regulatory B cells (Breg) in parallel with significantly lower IL-10 expression levels of CD24^+^CD27^+^ and CD24^high^CD38^high^ Breg in 2^nd^ HBvac non-responders compared to 2^nd^ HBvac responders. Anti-HBs seroconversion accompanied by a decrease of Breg numbers after booster immunization with a third-generation HBV vaccine, which indicates a positive effect of third-generation HBV vaccines on Breg-mediated immunomodulation in HBV vaccine non-responders.

The level of serum HBcAb has been demonstrated as a new marker of the responsiveness to antiviral treatment in CHB patients. Lou et al. showed that lower pre-transplantation level of HBcAb in patients undergoing liver transplantation is associated with HBV re-infection, suggesting that the baseline concentration of HBcAb may be a promising predictor for HBV recurrence in liver transplantation.

HBV mouse models have been widely used in studies on infection, immune responses, pathogenesis, and antiviral therapies. Du et al. summarized the currently available mouse models for HBV research. The HDI model is extensively used in various studies investigating immune responses to HBV, viral clearance, and evaluating novel antiviral agents. The AAV transduction-mediated replicon delivery model can be used in evaluating antiviral compounds, while controversial in the role in investigating HBV immunology. HBV transgenic mouse models are highly useful in elucidating the molecular virus-host interactions and HBV-related immunology and pathogenesis. Compared to other HBV mouse models, human liver chimeric mouse models possess obvious advantages, however, is limited due to the high cost and technical difficulties.

Taken together, the papers collected in this Research Topic covered a wide range of subjects in HBV research including the functional status of innate immunity, especially the PRRs, and HBV-specific T-B cells, IFN-based therapies, HBV-specific CAR-T/TCR-T, and target activation of PRRs, as well as the animal models for HBV infection. This collection reflects the current activities in a very important research field about the immunopathogenesis and immunotherapy of chronic HBV infection.

## Publisher’s Note

All claims expressed in this article are solely those of the authors and do not necessarily represent those of their affiliated organizations, or those of the publisher, the editors and the reviewers. Any product that may be evaluated in this article, or claim that may be made by its manufacturer, is not guaranteed or endorsed by the publisher.

## Author Contributions

MZ was a guest associate editor of the Research Topic and wrote the paper text. LJ was a guest associate editor of the Research Topic and edited the text, and acted as a coauthor or one paper in the Research Topic. AK is a guest associate editor of the Research Topic and edited the text, and acted as a coauthor of one paper in the Research Topic. LM was a guest associate editor of the Research Topic and edited the text, and acted as corresponding author for one paper in the Research Topic. All authors contributed to the article and approved the submitted version.

## Funding

MZ is supported by the National Science and Technology Major Project (2018ZX10723203, 2018ZX10302206, and 2017ZX10202202), the Foundation for Innovative Research Groups of Hubei Provincial Natural Science Foundation (2018CFA031), and Hubei Province’s Outstanding Medical Academic Leader Program. LJ is funded by the National Natural Science Foundation of China (82172256, 92169105, 81861138044 and 91742114). ADK is funded by the German Center for Infection research (DZIF) *via* project TTU05.803 “HBV Cure”.

## Conflict of Interest

The authors declare that the research was conducted in the absence of any commercial or financial relationships that could be construed as a potential conflict of interest.

## Publisher’s Note

All claims expressed in this article are solely those of the authors and do not necessarily represent those of their affiliated organizations, or those of the publisher, the editors and the reviewers. Any product that may be evaluated in this article, or claim that may be made by its manufacturer, is not guaranteed or endorsed by the publisher.

